# Origins of the amphiploid species *Brassica napus *L. investigated by chloroplast and nuclear molecular markers

**DOI:** 10.1186/1471-2229-10-54

**Published:** 2010-03-29

**Authors:** Charlotte J Allender, Graham J King

**Affiliations:** 1 Warwick HRI, University of Warwick, Wellesbourne, Warwick, CV35 9EF, UK; 2 Rothamsted Research, Harpenden, Hertfordshire, AL5 2JQ, UK

## Abstract

**Background:**

The amphiploid species *Brassica napus *(oilseed rape, Canola) is a globally important oil crop yielding food, biofuels and industrial compounds such as lubricants and surfactants. Identification of the likely ancestors of each of the two genomes (designated A and C) found in *B. napus *would facilitate incorporation of novel alleles from the wider *Brassica *genepool in oilseed rape crop genetic improvement programmes. Knowledge of the closest extant relatives of the genotypes involved in the initial formation of *B. napus *would also allow further investigation of the genetic factors required for the formation of a stable amphiploid and permit the more efficient creation of fully fertile re-synthesised *B. napus*. We have used a combination of chloroplast and nuclear genetic markers to investigate the closest extant relatives of the original maternal progenitors of *B. napus*. This was based on a comprehensive sampling of the relevant genepools, including 83 accessions of A genome *B. rapa *L. (both wild and cultivated types), 94 accessions of *B. napus *and 181 accessions of C genome wild and cultivated *B. oleracea *L. and related species.

**Results:**

Three chloroplast haplotypes occurred in *B. napus*. The most prevalent haplotype (found in 79% of accessions) was not present within the C genome accessions but was found at low frequencies in *B. rapa*. Chloroplast haplotypes characteristic of *B. napus *were found in a small number of wild and weedy *B. rapa *populations, and also in two accessions of cultivated *B. rapa *'brocoletto'. Whilst introgression of the *B. napus *chloroplast type in the wild and weedy *B. rapa *populations has been proposed by other studies, the presence of this haplotype within the two brocoletto accessions is unexplained.

**Conclusions:**

The distribution of chloroplast haplotypes eliminate any of the C genome species as being the maternal ancestor of the majority of the *B. napus *accessions. The presence of multiple chloroplast haplotypes in *B. napus *and *B. rapa *accessions was not correlated with nuclear genetic diversity as determined by AFLPs, indicating that such accessions do not represent recent hybrids. Whilst some chloroplast diversity observed within *B. napus *can be explained by introgression from inter-specific crosses made during crop improvement programmes, there is evidence that the original hybridisation event resulting in to *B. napus *occurred on more than one occasion, and involved different maternal genotypes.

## Background

*Brassica napus *(rapeseed, oilseed rape, Canola) is an oilseed crop of global economic significance. Over 50 million tonnes of rapeseed were produced in 2007 from an area of 30 million hectares [1]. In addition both tuberous (swede or rutabaga) and leafy forms (fodder rape and kale) of the species are grown as vegetables for human consumption and animal fodder. Oilseed *B. napus *has only achieved economic importance in the past forty years following an intensive breeding programme to decrease nutritionally undesirable components of the oil and meal, and to increase yields. Attention initially focused on reducing levels of erucic acid in the seed oil, and then reducing levels of aliphatic glucosinolate in the meal to make it more palatable and safer for livestock. More recently, varieties yielding oils suitable for conversion to biodiesel and industrial lubricants have been developed. As with other crops, ongoing breeding programmes aim to increase overall harvestable yield and quality, with resistance to crop pests and pathogens as major targets. Whilst successful, the collateral effect of these improvements has been the production of elite varieties that possess only a fraction of the genetic diversity available in the wider *Brassica *genepools. This is causing increasing concern, particularly with respect to lack of resistance to insect and other pests. Sources of new alleles that can easily be transferred into elite breeding lines are required in order to maintain and increase yield, provide new functional and adaptive disease resistance loci, and refine oil qualities to serve a variety of nutritional and industrial purposes.

The relationships between the six major cultivated *Brassica *species were originally described by U [2], who associated the diploid species *B. rapa*, *B. oleracea *and *B. nigra *L. with the amphiploids *B. juncea *L., *B. carinata *A. Br. and *B. napus*. Each of the amphiploids contains a combination of two diploid genomes. *B. napus *(n = 19) contains both the A and C genomes of its two progenitors, *B. rapa *(A genome, n = 10) and *B. oleracea *and related wild species (C genome, n = 9). Recreation of stable *B. napus *by crossing *B. rapa *and *B. oleracea *is difficult, but possible. Re-synthesized *B. napus *may be generated by crossing both diploid and tetraploid *B. rapa *and *B. oleracea*, although only a very small proportion of the attempted pollinations result in a viable hybrid plant [3]. In a current research context, the production of hybrids from diploid parents is commonly enhanced through embryo rescue or somatic hybridisation [4] although such plants often have a relatively high frequency of chromosomal rearrangements and reduced fertility [5,3].

The nature, direction and geographic location of the initial hybridisation events that led to the generation of *B. napus *remain unclear. *B. napus *is thought to be a relatively new species, since the earliest reliable documented record appears only 500 years ago, and although feral populations are common, no truly wild populations have been recorded ([6] and references therein). The location or locations of the original hybridisations is also unclear, as is whether they occurred in a wild or domesticated context. Both *B. rapa *and C genome species (particularly *B. oleracea*) have wide geographic ranges and geographically distinct centres of diversity. The earliest molecular studies suggested that the maternal parent of *B. napus *was likely to be *B. oleracea*, due to similarities in restriction patterns of their chloroplast genomes [7]. Subsequent RFLP analysis indicated that *B. montana*, a C genome relative of *B. oleracea*, had an identical chloroplast type to *B. napus*, and supported the contention that the maternal parent was not A genome *B. rapa *[8].

The identities of the A and C genome subtaxa involved in the original hybridisation that led to the formation of *B. napus *are not known, although [9] suggested that the genotype of the original A genome parent could be closely related to that of a *B. rapa *accession 'Spring Broccoli Raab'. However, the authors noted that post speciation introgression of *B. napus *genome fragments into the *B. rapa *accession could also explain their findings. Other studies have detected introgression of *B. rapa *into different *B. napus *genotypes [10]. Evidence based on either chloroplast or nuclear markers has suggested that *B. napus *appears to have resulted from several independent hybridisation events [9,8]. More recently, diversity in the *Brassica *chloroplast genome was characterised using nine microsatellite markers [11]. The study found 10 different haplotypes in the 15 *B. napus *individuals tested, although none of these haplotypes were shared by any other A or C genome species. Whilst of interest, such studies are restricted in value due to limited sampling and use of different marker systems that makes direct comparison or compilation impossible.

In order to establish a more robust basis for clarifying the origins of *B. napus*, and in particular to ascertain the species which was the likely maternal parent, we used both nuclear and chloroplast molecular markers. It was hoped that this approach would also provide baseline evidence to clarify the possible polyphyletic origins of the species. We carried out a detailed sampling of the genepools of the potential A and C genome donor species by surveying a total of 367 accessions representing 15 species. This included 94 *B. napus *accessions, together with 10 accessions of *B. montana*, the putative maternal ancestor of *B. napus *[8]. Representatives of *B. nigra *(B genome, n = 8) and the amphiploids *B. carinata *(BC genome, n = 17) and *B. juncea *(AB genome n = 18) were also included for comparison.

## Methods

In total we sampled 198 accessions representing 6 *Brassica *species (*B. napus*, *B. rapa*, *B. carinata*, *B. juncea*, *B. nigra *and *B. montana*) in order to determine diversity using chloroplast SSRs. Data are directly comparable with the 171 samples of *B. oleracea *and related C genome species previously described in [12]. Where possible, we selected accessions that had already been used in other published studies, particularly with regard to the *B. napus *and *B. rapa *accessions used in [8]. The *B. napus *accessions represent all *B. napus *crop types, with an emphasis on oilseeds due to their prevalence in an agricultural setting (global area sown and opportunities for gene flow to populations of related wild species). DNA was extracted from young leaves of a single seedling or single seeds as described in [12]. Most accessions were represented by a single sample. Tests on three individuals of five different *B. montana *accessions failed to detect more than one haplotype per accession (data not shown). Although it is likely that intra-accession chloroplast polymorphism is present, particularly within wild accessions, this is compensated by the large number of accessions sampled. Six primer pairs were used to amplify chloroplast SSRs, with PCR products visualised and scored on an ABI 3100 Genetic Analyzer (Applied Biosystems) following the methods described in [12].

A subset of 93 accessions were analysed for nuclear genome diversity using AFLPs using the restriction enzymes *EcoR*I and *Mse*I. This subset was chosen to be representative of the chloroplast haplotypes detected using the SSRs, whilst including a more thorough representation of particular groups of interest such as *B. rapa *brocoletto and *B. montana*. The number of accessions was limited to 93 in this analysis to avoid potential problems when combing data derived from different PCR reactions and to allow for control samples. Methods were based on those described by [13] except that we used a fluorescent detection system. A pre-selective step was carried out using the primers 5'-GACTGCGTACCAATTCA-3'and 5'-GATGAGTCCTGAGTAAC-3'which anneal to the *EcoR*I and *Mse*I adapters respectively. Selective amplifications with two primer pairs were then carried out, the primer sequences being identical to the pre-selective pairs with the addition of *EcoR*I-AAC-3'/*Mse*I-CAG-3'and *EcoR*I-AAG-3'/*Mse*I-CAA-3'. The *EcoR*I selective primer was labelled with FAM at the 5'end. Fragments were sized using an ABI 3100 Genetic Analyzer (Applied Biosystems) with a Genescan Rox 500 internal size standard and then scored using GeneMarker (Softgenetics) software. We examined peak heights across each trace and any peaks with a height less than the mean were regarded as absent. Traces from samples which had an overall mean peak height <200 relative fluorescence units (rfu) were disregarded in order to ensure only high quality data were analysed. As a control, AFLP analysis was carried out on six replicates of the same *B. nigra *sample to assess the robustness of the data.

**Figure 1:**
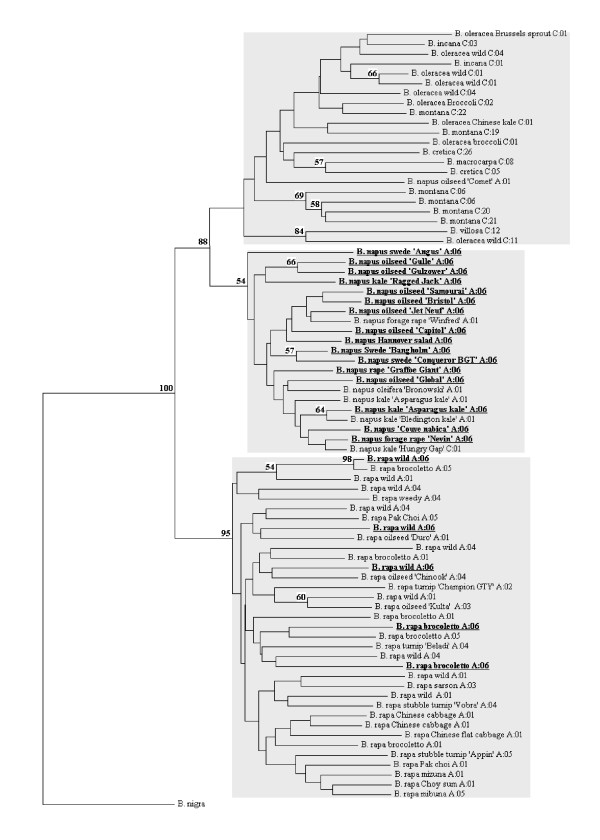
**NJ tree of AFLP data**. Chloroplast halptypes of each accession are indicated. Accessions with the A:06 (common *B. napus*) chloroplast haplotype are shown in bold and underlined. Numbers at nodes indicate % bootstrap support (out of 100 bootstrap replicates).

The diversity of *B. rapa *and *B. napus *was assessed using Nei's measure of gene diversity H [14]) based on the frequencies of the haplotypes present in each species. The AFLP data were analysed using Principal Coordinates Ordination (PCO) as implemented in the programme PAST (Øyvind Hammer and David Harper, available from http://folk.uio.no/ohammer/past/) using the DICE similarity metric. Nei's H was also calculated for *B. rapa*, *B. oleracea *and related C genome species, and *B. napus *using AFLP-SURV [15]. A Neighbour-Joining tree (Figure 1) based on the genetic distance measure of Link *et al*. [16] was constructed from the AFLP data using the software package TreeCon [17]. Support for the tree was assessed using 100 bootstrap replicates. Chloroplast haplotype data were also mapped onto this tree.

## Results

### Chloroplast Diversity

In total, 18 chloroplast haplotypes were resolved among the six *Brassica *species tested in this study. The sample details are given in Additional File 1 and the hapolotypes in Additional File 2. Combined with haplotypes previously established for C genome species accessions [12], a total of 38 haplotypes were present in the 367 *Brassica *accessions tested (Table 1). *B. rapa *exhibits a relatively high level of chloroplast polymorphism with H = 0.61. *B. napus *on the other hand is more diverse than *B. oleracea *(as reported in [12], H = 0.07) and with only 3 haplotypes detected in 94 accessions, we calculated H = 0.33.

**Table 1:** Species tested in this study or in Allender et al. (2007) and the chloroplast haplotypes detected using 6 SSRs.

Species	n	Haplotypes detected	Chloroplast SSR Haplotypes (n)
*B. napus*	94	3	A:01 *(16)*, A:06 *(75)*, C:01 *(3)*
*B. rapa*	88	6	A:01 *(46)*, A:02 *(2)*, A:03 *(4)*, A:04 *(15)*, A:05 *(16)*, A:06 *(5)*
*B. montana*	10	7	C:01 *(1)*, C:06 *(3)*, C:19 *(1)*, C:20 *(2)*, C:21 *(1)*, C:22 *(1)*, C:23 *(1)*
^†^*B. oleracea*	106	4	C:01 *(102)*, C:02 *(1)*, C:04 *(2)*, C:11 *(1)*
*B. juncea*	4	1	A:05 *(4)*
*B. carinata*	4	2	B:01 *(2)*, B:02 *(2)*
^†^*B. bourgaei*	1	1	C:01 *(1)*
^†^*B. cretica*	9	6	C:01 *(2)*, C:05 *(3)*, C:15 *(1)*, C:24 *(1)*, C:26 *(1)*, C:27 *(1)*
^†^*B. hilarionis*	4	2	A:01 *(3)*, C:25 *(1)*
^†^*B. incana*	11	2	C:01 *(10)*, C:03 *(1)*
^†^*B. insularis*	4	3	C:17 *(2)*, C:13 *(1)*, C:14 *(1)*
^†^*B. macrocarpa*	16	4	C:01 *(1)*, C:08 *(12)*, C:16 *(2)*, C:18 *(1)*
^†^*B. rupestris*	4	3	C:01 *(1)*, C:08 *(2)*, C:09 *(2)*
^†^*B. villosa*	13	6	C:01 *(1)*, C:07 *(11)*, C:09 *(3)*, C:10 *(1)*, C:11 *(4)*, C:12 *(3)*
*B. nigra*	3	1	B:01 *(3)*

^† ^Indicates data taken from Allender et al. (2007)

The three amphiploid species all possess chloroplast haplotypes characteristic of either of their respective progenitors (as originally proposed by [1]). *B. juncea *shares haplotype A:05 with *B. rapa*, whilst *B. carinata *and *B. nigra *share haplotype B:01. Three haplotypes (A:01, A:06 and C:01) are present amongst *B. napus *accessions with A:06 being the most prevalent (75 out of 94 accessions). This haplotype was also shared by two *B. rapa *(ssp. *ruvo *- crop type brocoletto) accessions. Haplotype A:06 is not present in any of the 171 C genome (including *B. oleracea *and *B. montana*) accessions. The A:01 haplotype occurs primarily in kale and spring oilseed *B. napus *accessions, whilst haplotype C:01 is only found in three kale accessions.

### AFLP Diversity

Of the six *B. nigra *control samples tested, four resulted in an AFLP trace with average peak height >200 rfu. From a total of 102 bands only three were scored differently between the four individuals, resulting in an overall fingerprint reproducibility of 97.1%. In total, 83 samples generated AFLP traces meeting the quality criterion. We calculated the number of polymorphic bands including the data from *B. nigra *(Table 2). The *B. nigra *samples contained 8 markers which were monomorphic among the A and C genome species tested. The two AFLP primer pairs yielded a total of 102 polymorphic bands across 83 accessions. *B. napus *had the highest mean number of bands per sample at 35.9, compared to *B. rapa *and the C genome species.

**Table 2:** AFLP summary statistics for the species groups tested; C genome species include *B. oleracea *and wild related species.

Species Group	n	Mean Bands present per sample	Diversity (H)
C genome	21	26.4	0.191
*B. rapa*	36	26.6	0.155
*B. napus*	22	35.9	0.111
*B. nigra*	*(4)*	*23.2*	*0.018*

**Figure 2:**
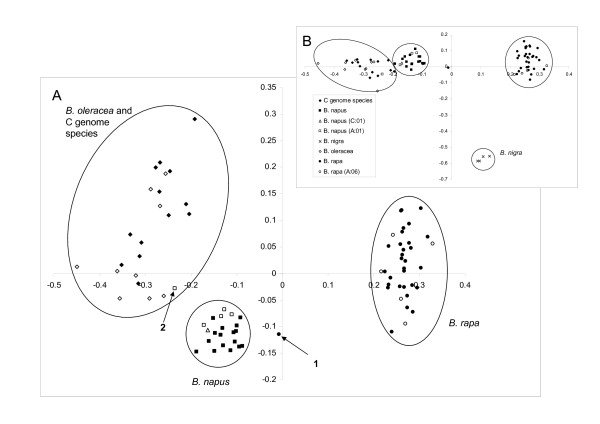
**Plot of first and second eigenvalues of the AFLP data**. Species clusters are identified along with samples with an atypical chloroplast haplotype. A - without *B. nigra *sample, B - with *B. nigra *sample to show the relative separation of the species clusters.

The PCO analysis based on the DICE similarity metric where the *B. nigra *replicates were included revealed that the first three eigenvalues explained 53.8% of the variation. The PCO plot (Figure 2) shows that the *B. napus*, *B. rapa *and C genome species fall into well defined clusters, with only two exceptions. We carried out the PCO both excluding (Figure 2a) and including (Figure 2b) the *B. nigra *samples. Omitting the *B. nigra *samples had the effect of increasing the resolution of the PCO due to the relative differences in genetic distance between the A and C genome species and B genome *B. nigra*. The C genome accessions (comprising several different but closely related species) are more loosely grouped than both the *B. rapa *and *B. napus *samples, indicating the greater genetic diversity within the C genome species. The two accessions falling outside of the species clusters include one *B. rapa *('1' on Figure 2a) sampled from a weedy population located in a *B. napus *oilseed rape field in the UK, and an accession ('2') sampled from a spring oilseed *B. napus *variety 'Comet'. For both *B. rapa *and *B. napus*, samples with haplotypes more commonly found in other species are found within the conspecific cluster - they are not distinguishable by the AFLP analysis.

The neighbour-joining tree we constructed from these data (Figure 1) clusters accessions into species groups with relatively high levels of bootstrap support. All *B. rapa *accessions group together. All *B. napus *accessions (kale, swede and oilseed) also cluster, although there is very little in the way of internal structure within this group. The branch lengths in the *B. napus *group also appear to be shorter than for other species, indicating less intra-specific differentiation. This shows that although more markers are amplified in the *B. napus *samples, there is less variability in these markers. The situation is slightly more complex for the C genome species. Four of the six *B. montana *accessions form a well defined group within the other C genome accessions with 69% bootstrap support, whilst the other two accessions are distributed more widely within the C genome group.

## Discussion

The chloroplast haplotypes found in *B. napus *effectively rule out most of the C genome species from the maternal lineage of nearly all of the *B. napus *samples tested. Only three samples (all kale types) had a haplotype commonly associated with *B. oleracea*. A small number of *B. napus *samples shared haplotype A:01 with both *B. rapa *and *B. hilarionis*. The most prevalent haplotype in *B. napus *(A:06) occurs elsewhere in only a small number of *B. rapa *samples. Three of these A:06 *B. rapa *accessions are from the UK and were collected from wild or weedy populations occurring within or alongside *B. napus *oilseed rape fields. The remaining two samples are 'brocoletto' types from Italy. There are two explanations for the co-occurrence of A:06 in *B. rapa *and *B. napus*. One is that the original donor of A:06 was not sampled in this study, and that any occurrence of A:06 in *B. rapa *is the result of recent or historical introgression from *B. napus *into *B. rapa*. An introgressed origin of the A:06 chloroplast is suggested as this haplotype is more common in UK populations which exist in close proximity to oilseed rape [18]. Additionally, *B. napus *was historically grown very widely in the UK in the 19^th ^Century as a fodder crop (swedes and turnips occupied at least 598 k ha in England in 1881; [19]), providing further opportunity for introgression.

We sampled most of the geographical and morphological diversity of *B. rapa*. Outside of the UK, haplotype A:06 only occurred in two of the eight brocoletto accessions tested. The AFLP analysis shows that these accessions are not recent hybrids with *B. napus *or *B. oleracea *since they cluster as expected with the rest of the *B. rapa *samples in the PCO plot and the NJ tree (figures 1 and 2). The AFLP analysis is sensitive to inter-specific hybrids as demonstrated by the single weedy *B. rapa *individual ('1' on Figure 2a - chloroplast haplotype A:04) which falls between the *B. rapa *and *B. napus *clusters in the PCO plot. We tested this *B. rapa *sample further with three nuclear CAPS (cleaved amplified polymorphic sequence) markers and three nuclear SSRs. These markers revealed the presence of both A and C genome alleles (data not shown). Interestingly, the three UK wild/weedy individuals with the A:06 haplotype do not have C genome markers as tested by the AFLPs. They also cluster with the rest of the *B. rapa *accessions. We conclude that either the introgression event must have been followed by many generations of back crossing to *B. rapa*, or that the C genome fragments were lost rapidly within a few generations.

The relatively recent origin of *B. napus *as a species is supported by the reduced diversity as determined by Nei's measure of gene diversity within *B. napus*, compared to the A and C genome ancestral genepools. It is difficult at present to be certain whether the original hybridisation event(s) involved more than one chloroplast haplotype. However, multiple hybridisation events are indicated by the presence of three different cytoplasms among the *B. napus *samples tested. Again, this could alternately be explained by post-speciation introgression. This is indeed the case for some spring oilseed types and fodder rape types where *B. rapa *and *B. oleracea *are documented to have been used in *B. napus *breeding programmes [3,20]. A:01 and C:01 haplotypes also occur in non-oilseed *B. napus *crop types, namely rape kales and fodder kales, where crop improvement programmes may also at least be responsible in part for their presence. However, only two accessions of these kale and fodder types with A:01 or C:01 chloroplasts are classed as 'advanced cultivars' (i.e. resulting from a formal modern crop improvement programme). The remainder are 'traditional varieties or landraces' so are less likely to have been developed from deliberate inter-specific crossing. As with *B. rapa*, the AFLP analysis does not differentiate between the different cytoplasmic types of *B. napus*, with a single exception. A sample from the spring oilseed variety 'Comet' (A:01 haplotype) falls outside of the well defined cluster of *B. napus *samples, and in fact groups within the C genome cluster. The reasons for this are unclear and further testing would be required for confirmation of this result. The presence of haplotype A:01 in *B. hilarionis *as reported by [12] is intriguing and may shed light on the common ancestry of A and C genome species. Although it is possible that *B. hilarionis *represents a source of the A:01 chloroplast in *B. napus *this is unlikely since *B. hilarionis *is endemic to Cyprus.

The *B. nigra *sample included in the AFLP analysis provided both an outgroup for clustering analysis and a control. Overall reproducibility of the five replicates was 97.1% as only three bands out of a total of 102 were scored differently. The chloroplast haplotype (B:01) found in the three different *B. nigra *accessions is very distinct from those found in the A or the C genome, and the PCO analysis of AFLP data shows that the other *Brassica *samples tested are more similar to each other than they are to *B. nigra*. This in agreement with the findings of [21], in their investigation of the phylogeny of the Brassicaceae.

The tree constructed from the AFLP data shows good discrimination between *B. napus*, the C genome species, and *B. rapa*. However, intra-specific relationships remain mostly unclear. This is probably due to the relatively low marker to sample ratio (102:83). Future studies may improve resolution through the use of massively parallel sequencing technologies rather than the anonymous markers produced using AFLP. However, intra-specific relationships were not the primary focus of this study, and the PCO and NJ tree clearly indicate that intra-specific cytoplasmic differences are not always associated with whole genome diversity.

In contrast with [8], we did not find that any of the ten *B. montana *accessions tested shared a chloroplast haplotype with *B. napus*. We were not able to test exactly the same accession used by Song and Osborn as no further seed was available. A further '*B. montana*' accession (not the same as that used by Song and Osborn) was originally included in our study. However subsequent taxonomic verification based on plant morphology revealed it to be incorrectly identified, and indeed it was very similar in appearance to *B. napus*. The AFLP data for this accession also indicated it was *B. napus*. Even though none of our *B. montana *accessions shared the A:06 chloroplast haplotype with *B. napus*, three of them did possess the next most closely related haplotype (C:06 - see [12]). It is possible that the RFLP markers used by [8] did not distinguish between these chloroplast genomes.

The *B. oleracea *chloroplast type has been detected previously in another *B. napus *accession, 'New Zealand Rawara' and this was proposed as further evidence to support a polyphyletic origin for *B. napus *[8]. We tested the ploidy level of one individual of this accession using flow cytometry, and discovered that it was in fact *B. oleracea*. However, three other verified *B. napus *samples in our study did contain the C:01 chloroplast common in *B. oleracea*. We did not find more than one of the *B. rapa *chloroplast haplotypes in *B. napus*, unlike [8] who detected two. Our sampling strategy was based on maximising the coverage of the A and C genome genepools by only testing one individual per accession. As with material derived from most *ex situ *genetic resource collections, many of the accessions are sampled from wild populations, local selections and open-pollinated varieties, and as such one would expect a degree of variation within accessions. In addition to cases of mis-identification as demonstrated above, there is always potential for apparent differences between studies to result from within-accession variation arising either from natural diversity or contamination of seed lots. We minimised these factors through using either ploidy analysis or by visual taxonomic confirmation of plants. Confirmation of the taxomomy of accessions will be facilitated in future by the ongoing efforts in genetic resource collections to provide online visual records of mature plants.

Multiple hybridisation events consistent with a polyphyletic origin were also indicated by the results of [9] who found that a sample of *B. napus *'asparagus kale' differed in RFLP profile from other *B. napus *tested, suggesting an additional diploid parental genotype. We also found that five out of the six asparagus kale accessions in our study had the A:01 chloroplast haplotype typical of *B. rapa*. Most of these are traditional varieties and unlikely to have been selected through formal crop improvement. Such evidence suggests that *B. napus *may indeed have multiple origins. In addition, [9] also found that a *B. rapa *accession ('spring broccoli raab' - another name for the brocoletto crop type) shared a unique marker with the majority of *B. napus *samples in their study. This marker was absent from all other potential diploid progenitors, including *B. oleracea*. Interestingly, the spring broccoli raab sample tested was the only *B. rapa *to possess markers more commonly associated with the C genome. The authors suggest that the presence of (presumably introgressed) C genome fragments may have facilitated the inter-specific hybridisation which lead to the formation of a stable *B. napus*.

A recent study also based on chloroplast SSRs did not find any haplotypes in common between *B. napus*, *B. oleracea *and *B. rapa *[11]. Five varieties of *B. napus *were tested using nine SSRs and the authors detected eleven unique haplotypes, indicating that their nine markers detected a much higher level of intra-accession diversity than our six. Since the *B. napus *haplotypes were much more similar to those found in *B. rapa *than *B. oleracea*, the authors suggested that *B. rapa *was a much more likely maternal progenitor for *B. napus *than *B. oleracea*. This supports the findings of our study. However, as the authors indicated, SSR markers mutate at a relatively high rate, leading to the possibility of homoplasy and parallel origins of allele size. This, in addition to the hybridized origins of a significant portion of *Brassica *breeding material means that chloroplast SSRs alone may not provide sufficient information for conclusions to be drawn on maternal ancestry.

Artificial (re-synthesised) *B. napus *is known to undergo a relatively high frequency (compared to natural *B. napus*) of genomic rearrangements, including non-reciprocal translocations, due to pairing between homeologous chromosomes at meiosis [5]. Evidence has accumulated through several studies that a genetic factor regulating chromosome pairing is present in *B. napus *[22]. Control of chromosome pairing is required in order prevent the formation of unbalanced gametes and aneuploid progenies which reduced fertility. Identification of the closest extant relatives of the original A and C genome genotypes involved in the initial hybridisations leading to *B. napus *should allow closer investigations of these mechanisms and enable the resynthesis of a more meiotically stable artificial *B. napus*.

## Conclusions

Our study indicates that it is highly unlikely that *B. oleracea *or any of the C genome species are closely related to the maternal progenitor of most *B. napus *accessions. The detection of two other chloroplast SSR haplotypes at low frequencies in *B. napus *does suggest that multiple hybridisation events involving different maternal ancestors may have occurred. However, the use of inter-specific hybrids (and re-synthesised *B. napus*) in modern crop improvement programmes is most likely responsible for some of the observed diversity. Natural post-speciation introgression (or chloroplast capture) is also a possibility since *B. napus *(A:06) chloroplasts are observed at a frequency of 0.12 in some *B. rapa *populations sympatric with oilseed rape fields [18]. *B. napus *samples with 'atypical' cytoplasm are not usually distinguishable from those harbouring the prevalent chloroplast haplotype in terms of nuclear genome diversity. Our results are consistent with those of [9] who suggested that *B. rapa *'spring broccoli raab' may be the closest extant relative of the maternal ancestor of *B. napus*. Our study based on chloroplast haplotypes and 102 AFLP markers provides further support to their inference, based on 38 nuclear and 6 chloroplast RFLP probes, since the prevalent *B. napus *haplotype was also found in two additional accessions of the same crop type.

Given that no truly wild populations of *B. napus *have been documented, it seems reasonable to suggest that the initial hybridisations must have occurred in a cultivated context rather than a wild setting [6]. If the A:06 chloroplast haplotype detected in the two brocoletto accessions is not the result of chloroplast capture, then it is possible to envisage hybridisation occurring between brocoletto and *B. oleracea *crops growing in the same location. The brocoletto crop type originates from southern Italy, and a similar crop is also grown in Portugal. In both of these areas, it is highly likely that brocoletto would have been cultivated alongside *B. oleracea *crops such as kales, cabbages and broccolis, providing the necessary opportunities for inter-specific crosses to occur. Further work on the genetic diversity of the brocoletto crop type is required to verify such speculation.

Future genetic improvement of *B. napus *crops (e.g. focusing on abiotic stress tolerance, pest and disease resistance and other yield increases) will depend to a large degree on utilising the diversity present within the ancestral A and C genepools. It is clear that hybridisation between *B. rapa *and *B. oleracea *is very rare in nature, and knowing which genotypes of the parental species were involved will allow a greater understanding of the mechanisms and genetic factors controlling the creation of stable amphiploids, and this will facilitate the incorporation of novel alleles from the wider *Brassica *genepool.

## Authors' contributions

CJA conceived of the study, carried out the molecular marker work, analysed the data and drafted the manuscript. GJK provided intellectual input and assistance with drafting the manuscript. Both authors have read and approved the final manuscript.

## Authors' Information

Charlotte Allender is the Assistant Manager of the Genetic Resources Unit at Warwick HRI which maintains globally significant vegetable seed collections. Her research interests centre on the processes and partitioning of genetic diversity in crops and their wild relatives. Graham King has wide experience of quantitative genetics underlying crop trait improvement. His group at Rothamsted Research are involved in comparative genomics, epigenetics and developmental biology with a focus on seed development of oilseed brassicas. He leads the UK Oilseed Rape Genetic Improvement Network and his group host the http://www.brassica.info website.

## Supplementary Material

Additional file 1 **Table S1**. Details of the samples and accessions used in this study. 'ID' is a unique identifier for each sample, a * in the second column indicates data have been taken from [12]. The samples included in the AFLP analysis are identified. Click here for file

Additional file 2 **Table S2**. Allelic constitution of the new chloroplast haplotypes detected in this study using the 6 chloroplast SSRs as well as those detected in samples used for the AFLP analysis Click here for file
