# Supplementary material for: Origins of the amphiploid species Brassica napus L. investigated by chloroplast and nuclear molecular markers
Source: BMC Plant Biol. 2010 Mar 29;10:54. doi: 10.1186/1471-2229-10-54 (PMC2923528; doi:10.1186/1471-2229-10-54)
Supplement: Additional file 1 — Table S1. Details of the samples and accessions used in this study. 'ID' is a unique identifier for each sample, a * in the second column indicates data have been taken from [12]. The samples included in the AFLP analysis are identified. [file 1471-2229-10-54-S1.PDF]

Table S1. Details of the samples and accessions used in this study. 'ID' is a unique identifier for each sample, a * in the second column indicates data have been taken from Allender et al (2007). The samples included in the AFLP analysis are identified.

ID		AFLP	Species	Subspecies	description	Other name	Accession Number	Haplotype
alt1	*	-	Brassica atlantica	-	wild species C	C 04002	HRIGRU007339B	C:17
alt2	*	-	Brassica atlantica	-	wild species C	C 04003	HRIGRU012704	C:17
bou1	*	-	Brassica bourgaei	-	wild species C	C 04006	HRIGRU011452	C:01
car1		-	Brassica carinata	-	B. carinata	GK040947	GK040946	B:01
car2		-	Brassica carinata	-	B. carinata	GK040944	GK040947	B:02
car3		-	Brassica carinata	-	B. carinata	-	GK040942	B:02
car4		-	Brassica carinata	-	B. carinata	-	GK040945	B:01
cre1	*	-	Brassica cretica	-	wild species C	C 04013	CA 02001	C:19
cre2	*	-	Brassica cretica	-	wild species C	C 04014	CA 98004	C:05
cre3	*	-	Brassica cretica	-	wild species C	B. cretica	GK99123	C:01
cre4	*	-	Brassica cretica	-	wild species C	C 04007	HRIGRU007328	C:01
cre5	*	yes	Brassica cretica	-	wild species C	C 04008	HRIGRU007329	C:05
cre6	*	-	Brassica cretica	-	wild species C	C 04009	HRIGRU007334	C:22
cre7	*	yes	Brassica cretica	-	wild species C	C 04010	HRIGRU007335	C:21
cre8	*	-	Brassica cretica	-	wild species C	C 04011	HRIGRU008225	C:05
cre9	*	-	Brassica cretica	-	wild species C	C 04012	HRIGRU011462	C:15
hil1	*	-	Brassica hilarionis	-	wild species C	C 04015	HRIGRU011463	C:20
hil2	*	-	Brassica hilarionis	-	WILD SPECIES	ST HILARION CABBAGE	HRIGRU012483	A:01
hil3	*	-	Brassica hilarionis	-	wild species C	C 04016	HRIGRU012483B	A:01
hil4	*	-	Brassica hilarionis	-	wild species C	C 04017	HRIGRU012576	A:01
inc1	*	-	Brassica incana	-	wild species C	C 04023	CA 00003	C:01
inc10	*	-	Brassica incana	-	wild species C	C 04020	HRIGRU013140	C:01
inc11	*	-	Brassica incana	-	wild species C	C 04021	HRIGRU013141	C:01
inc2	*	-	Brassica incana	-	wild species C	B. incana	CA91087	C:01
inc3	*	-	Brassica incana	-	wild species C	C 04079	HRIGRU006691	C:01
inc4	*	-	Brassica incana	-	wild species C	C 04080	HRIGRU006849	C:01
inc5	*	-	Brassica incana	-	wild species C	C 04081	HRIGRU007324	C:01
inc6	*	-	Brassica incana	-	wild species C	C 04082	HRIGRU007330	C:01
inc7	*	-	Brassica incana	-	wild species C	C 04077	HRIGRU007345	C:01
inc8	*	yes	Brassica incana	-	wild species C	C 04018	HRIGRU007351	C:03
inc9	*	yes	Brassica incana	-	wild species C	C 04019	HRIGRU012484	C:01
ins1	*	-	Brassica insularis	-	wild species C	C 04024	HRIGRU007326	C:13
ins2	*	-	Brassica insularis	-	wild species C	C 04027	HRIGRU011449	C:14
jun1		-	Brassica juncea	-	brown mustard	-	HRIGRU004266	A:05
jun2		-	Brassica juncea	-	brown mustard	-	HRIGRU004272	A:05
jun3		-	Brassica juncea	-	brown mustard	-	HRIGRU004277	A:05

jun4		-	Brassica juncea	-	brown mustard	-	HRIGRU004304	A:05
mac1	*	-	Brassica macrocarpa	-	wild species C	B. macrocarpa	CA91064	C:01
mac10	*	-	Brassica macrocarpa	-	wild species C	C 04037	HRIGRU013119	C:08
mac11	*	-	Brassica macrocarpa	-	wild species C	C 04038	HRIGRU013120	C:08
mac12	*	-	Brassica macrocarpa	-	wild species C	C 04039	HRIGRU013121	C:08
mac13	*	-	Brassica macrocarpa	-	wild species C	C 04040	HRIGRU013122	C:08
mac14	*	yes	Brassica macrocarpa	-	wild species C	C 04041	HRIGRU013123	C:08
mac15	*	-	Brassica macrocarpa	-	wild species C	C 04042	HRIGRU013124	C:08
mac16	*	-	Brassica macrocarpa	-	wild species C	C 04043	HRIGRU013125	C:08
mac2	*	-	Brassica macrocarpa	-	wild species C	C 04028	HRIGRU007327	C:16
mac3	*	-	Brassica macrocarpa	-	wild species C	C 04030	HRIGRU012703	C:08
mac4	*	-	Brassica macrocarpa	-	wild species C	C 04031	HRIGRU013112	C:08
mac5	*	-	Brassica macrocarpa	-	wild species C	C O4032	HRIGRU013113	C:08
mac6	*	-	Brassica macrocarpa	-	wild species C	C 04033	HRIGRU013114	C:16
mac7	*	-	Brassica macrocarpa	-	wild species C	C 04034	HRIGRU013116	C:18
mac8	*	-	Brassica macrocarpa	-	wild species C	C 04035	HRIGRU013117	C:08
mac9	*	-	Brassica macrocarpa	-	wild species C	C 04036	HRIGRU013118	C:08
mon1	*	-	Brassica montana	-	wild species C	C 04070	HRIGRU007337	C:06
mon10		-	Brassica montana	-	wild species C	GK040958	UPM 9864	C:20
mon2	*	yes	Brassica montana	-	wild species C	C 04045	HRIGRU011466	C:06
mon3	*	yes	Brassica montana	-	wild species C	C 04071	HRIGRU012702	C:06
mon4		yes	Brassica montana	-	wild species C	GK040951	UPM 5979	C:19
mon5		-	Brassica montana	-	wild species C	GK040952	UPM 6801	C:01
mon6		yes	Brassica montana	-	wild species C	GK040953	UPM 6813	C:22
mon7		-	Brassica montana	-	wild species C	GK040954	UPM 6815	C:23
mon8		yes	Brassica montana	-	wild species C	GK040955	UPM 6817	C:21
mon9		yes	Brassica montana	-	wild species C	GK040956	UPM 9860	C:20
nap1		-	Brassica napus	oleifera biennis	Winter oilseed rape	Ningyou 7	-	A:06
nap10		-	Brassica napus	oleifera biennis	Winter oilseed rape	RafalBAZ	BAZ 34411	A:06
nap11		-	Brassica napus	oleifera biennis	Winter oilseed rape	MatadorBAZ	BAZ 34425	A:06
nap12		yes	Brassica napus	oleifera annua	Spring oilseed rape	GulzowerBAZ	BAZ 34431	A:06
nap13		-	Brassica napus	oleifera annua	Spring oilseed rape	NiklasBAZ	BAZ 49358	A:06
nap14		-	Brassica napus	oleifera annua	Spring oilseed rape	KaratBAZ	BAZ 49359	A:06
nap15		-	Brassica napus	oleifera biennis	Winter oilseed rape	ElenaBAZ	BAZ 58381	A:06
nap16		-	Brassica napus	oleifera annua	Spring oilseed rape	HermesBAZ	BAZ 65334	A:01
nap17		-	Brassica napus	oleifera biennis	Winter oilseed rape	LictorBAZ	BAZ 69475	A:06
nap18		-	Brassica napus	oleifera biennis	Winter oilseed rape	QuintaBAZ	BAZ 7302	A:06

nap19		yes	Brassica napus	pabularia	Hannover Salad?	Brauner Schnittkohl	BRA 175	A:06
nap2		-	Brassica napus	oleifera annua	Spring oilseed rape	Brutor	15O0200095	A:06
nap20		-	Brassica napus	pabularia	Hannover Salad?	Grüner Schnittkohl	BRA 177	A:06
nap21		-	Brassica napus	oleifera biennis	Winter oilseed rape	PAK OSR	CGN07224	A:01
nap22		yes	Brassica napus	oleifera biennis	Winter oilseed rape	JetNeufCGN	CGN07227 24/3/00	A:06
nap23		-	Brassica napus	oleifera biennis	Winter oilseed rape	Shen-Li Jutsaj	CGN17305	A:06
nap24		-	Brassica napus	oleifera biennis	Winter oilseed rape	FalconCGN	CGN17323 23/10/95	A:06
nap25		-	Brassica napus	oleifera biennis	Winter oilseed rape	CeresCGN	CGN17385 23/10/95	A:06
nap26		-	Brassica napus	oleifera biennis	Winter oilseed rape	ArianaCGN	CGN18952 13/5/97	A:06
nap27		-	Brassica napus	oleifera biennis	Winter oilseed rape	BienvenuCGN	CGN18955 13/5/97	A:06
nap28		yes	Brassica napus	oleifera biennis	Winter oilseed rape	BristolCGN	CGN18957 13/5/97	A:06
nap29		-	Brassica napus	oleifera biennis	Winter oilseed rape	CapricornCGN	CGN18959 13/5/97	A:06
nap3		-	Brassica napus	oleifera annua	Spring oilseed rape	Stellar	15O0200150	A:01
nap30		-	Brassica napus	oleifera biennis	Winter oilseed rape	CobraCGN	CGN18960 13/5/97	A:06
nap31		yes	Brassica napus	oleifera biennis	Winter oilseed rape	SamouraiCGN	CGN18975 13/5/97	A:06
nap32		yes	Brassica napus	oleifera annua	Spring oilseed rape	GlobalCGN	CGN19967 22/1/98	A:06
nap33		-	Brassica napus	oleifera annua	Spring oilseed rape	HannaCGN	CGN19968 22/1/98	A:01
nap34		-	Brassica napus	oleifera annua	Spring oilseed rape	Westar	CR1054	A:06
nap35		-	Brassica napus	oleifera annua	Spring oilseed rape	Marnoo	CR765	A:06
nap36		-	Brassica napus	oleifera biennis	Winter oilseed rape	RocketCPB	GK020628	A:06
nap37		-	Brassica napus	oleifera biennis	Winter oilseed rape	LiptonCPB	GK0230002	A:01
nap38		-	Brassica napus	oleifera biennis	Winter oilseed rape	CanberraCPB	GK030001	A:06
nap39		-	Brassica napus	oleifera biennis	Winter oilseed rape	FortressCPB	GK030004	A:06
nap4		yes	Brassica napus	oleifera annua	Spring oilseed rape	BronowskiBAZ	BAZ 11945	A:01
nap40		-	Brassica napus	oleifera biennis	Winter oilseed rape	MadrigalCPB	GK030005	A:06
nap41		-	Brassica napus	oleifera biennis	Winter oilseed rape	EscortCPB	GK030006	A:06
nap42		-	Brassica napus	oleifera biennis	Winter oilseed rape	RecitalCPB	GK03003	A:06
nap43		-	Brassica napus	napobrassica	Swede	Acme	HRIGRU003243	A:06
nap44		-	Brassica napus	biennis	Winter forage rape	English Giant	HRIGRU003258	A:06
nap45		yes	Brassica napus	napobrassica	SWEDE	BANGHOLM	HRIGRU003277	A:06
nap46		yes	Brassica napus	biennis	WINTER FORAGE RAPE	NEVIN	HRIGRU003306	A:06
nap47		-	Brassica napus	biennis	Winter forage rape	Bishop	HRIGRU003308	A:06
nap48		yes	Brassica napus	biennis	WINTER FORAGE RAPE	WINFRED	HRIGRU003337	A:01
nap49		yes	Brassica napus	napobrassica	SWEDE	CONQUEROR BRONZE GREEN TOP	HRIGRU003470	A:06
nap5		yes	Brassica napus	oleifera annua	Spring oilseed rape	GulleBAZ	BAZ 16219	A:06
nap50		yes	Brassica napus	biennis	Rape	Graffoe Giant	HRIGRU004045	A:06
nap51		-	Brassica napus	oleifera	OILSEED (NAPUS!)	EMERALD 65	HRIGRU004570	A:06

nap52		yes	Brassica napus	biennis	RAPE KALE	RAGGED JACK	HRIGRU005671	A:06
nap53		-	Brassica napus	biennis	Rape kale	Asparagus kale	HRIGRU005673	A:01
nap54		yes	Brassica napus	biennis	Rape kale	Asparagus kale	HRIGRU005675	A:01
nap55		yes	Brassica napus	biennis	Rape kale	Bledington Kale	HRIGRU005676	A:01
nap56		-	Brassica napus	biennis	Rape kale	Black Jack	HRIGRU005681	C:01
nap57		-	Brassica napus	biennis	Rape kale	Jerusalem Kale	HRIGRU005684	A:01
nap58		-	Brassica napus	biennis	Rape Kale	Asparagus kale	HRIGRU005691	A:01
nap59		-	Brassica napus	biennis	Rape kale	Old fashioned greens	HRIGRU005691	A:06
nap6		-	Brassica napus	oleifera biennis	Winter oilseed rape	MajorBAZ	BAZ 28659	A:06
nap60		-	Brassica napus	oleifera biennis	RAPE	MOANA	HRIGRU005847	A:06
nap61		-	Brassica napus	napobrassica	SWEDE	DRUMMONDS PURPLE TOP	HRIGRU005878	A:06
nap62		-	Brassica napus	biennis	Rape kale	Asparagus kale	HRIGRU006224	A:01
nap63		-	Brassica napus	napobrassica	swede	KALFAFELLSROFA	HRIGRU006340	A:06
nap64		-	Brassica napus	unknown	B. napus 'wild'	?	HRIGRU006528	A:06
nap65		yes	Brassica napus	napobrassica	SWEDE	ANGUS	HRIGRU006627	A:06
nap66		-	Brassica napus	biennis	RAPE KALE	ASPARAGUS KALE	HRIGRU006692	A:06
nap67		yes	Brassica napus	napobrassica	SWEDE	TINA	HRIGRU006724	A:06
nap68		-	Brassica napus	napobrassica	SWEDE	ALTASWEET	HRIGRU006724	A:06
nap69		-	Brassica napus	biennis	WINTER FORAGE RAPE	CANARD	HRIGRU006804	A:06
nap7		-	Brassica napus	oleifera annua	Spring oilseed rape	OroBAZ	BAZ 28678	A:06
nap70		-	Brassica napus	napobrassica	SWEDE	LORD DERBY	HRIGRU006899	A:06
nap71		yes	Brassica napus	biennis	Kale	Hungry Gap	HRIGRU007553	C:01
nap72		-	Brassica napus	oleifera biennis	Winter oilseed rape	PrimorHRI	HRIGRU009138	A:06
nap73		-	Brassica napus	biennis	Rape kale	Russian Kale	HRIGRU009805	A:06
nap74		-	Brassica napus	biennis	Kale	-	HRIGRU012361	A:06
nap75		yes	Brassica napus	unknown	COUVE NABICA	COUVE NABICA	HRIGRU009522	A:06
nap76		-	Brassica napus	oleifera annua	Spring oilseed rape	TargetIPK	IPK CR 1014/96 BNS	A:06
nap77		-	Brassica napus	oleifera annua	Spring oilseed rape	TowerIPK	IPK CR 1025/93 BNS	A:06
nap78		-	Brassica napus	oleifera annua	Spring oilseed rape	GULZOWERIPK	IPK CR 637/85 BNS	A:06
nap79		-	Brassica napus	oleifera annua	Spring oilseed rape	LihoIPK	IPK CR 704/81 BNS	A:06
nap8		-	Brassica napus	oleifera annua	Spring oilseed rape	NuggetBAZ	BAZ 28680	A:06
nap80		-	Brassica napus	oleifera annua	Spring oilseed rape	ReginaIPK	IPK CR 882/86 BNS	A:01
nap81		-	Brassica napus	oleifera biennis	Winter oilseed rape	SareptaIPK	IPK CR 904/79 BNN	A:06
nap82		-	Brassica napus	oleifera annua	Spring oilseed rape	HANNANGB	NGB 13112.1	A:01
nap83		-	Brassica napus	oleifera annua	Spring oilseed rape	MarinkaNGB	NGB 1349.1	A:06
nap84		-	Brassica napus	oleifera biennis	Winter oilseed rape	GazelleNGB	NGB 13492.1	A:06

nap85		-	Brassica napus	oleifera biennis	Winter oilseed rape	HeraldNGB	NGB 13512.1	A:06
nap86		yes	Brassica napus	oleifera annua	Spring oilseed rape	CometNGB	NGB 13940.1	A:01
nap87		yes	Brassica napus	oleifera biennis	Winter oilseed rape	VictorNGB	NGB 590.1 1981	A:06
nap88		-	Brassica napus	oleifera biennis	Winter oilseed rape	LembkesRICP	RICP 1500100025	A:06
nap89		-	Brassica napus	oleifera biennis	Winter oilseed rape	MikadoRICP	RICP 1500100362	A:06
nap9		-	Brassica napus	oleifera biennis	Winter oilseed rape	LedosBAZ	BAZ 29621	A:06
nap90		-	Brassica napus	oleifera biennis	Winter oilseed rape	AsahiRICP	RICP 1500100411	A:06
nap91		-	Brassica napus	oleifera biennis	Winter oilseed rape	NavajoRICP	RICP 1500100465	A:06
nap92		yes	Brassica napus	oleifera biennis	Winter oilseed rape	CapitolRICP	RICP 1500100477	A:06
nap93		-	Brassica napus	oleifera biennis	Winter oilseed rape	EnvolRICP	RICP 1500100491	A:06
nap94		-	Brassica napus	napus	fodder rape	Akela	CGN07231	C:01
nig1		yes	Brassica nigra	-	wild species B	B. nigra	CA97089	B:01
nig2		-	Brassica nigra	-	B. nigra	-	HRIGRU010978	B:01
nig3		-	Brassica nigra	-	B. nigra	-	HRIGRU011011	B:01
ole1	*	-	Brassica oleracea	italica	broccoli	Marathon DH line Mar34	12479	C:01
ole10	*	-	Brassica oleracea	wild	Wild species C	Wild B. oleracea	GK010790/1	C:01
ole100	*	-	Brassica oleracea	botrytis	cauliflower	Roscoff type F1 DJ1356 DH line 152b	ROS152b	C:01
ole101	*	-	Brassica oleracea	italica	broccoli	Shogun DH line 5a	SHO5a	C:01
ole102	*	-	Brassica oleracea	botrytis	cauliflower	Siria DH line 5a	SIR5a	C:01
ole103	*	-	Brassica oleracea	botrytis	cauliflower	Surfrider DH line 186a	SUR186a	C:01
ole104	*	-	Brassica oleracea	botrytis	cauliflower	Woomera DH line 38a	WOM38a	C:01
ole105	*	yes	Brassica oleracea	wild	wild species C	C 04098	WS15	C:11
ole106	*	-	Brassica oleracea	alboglabra	wild species C	C 04001	HRIGRU011464	C:01
ole11	*	-	Brassica oleracea	capitata	cabbage	Hawke DH line Ha84a	HA 84a	C:01
ole12	*	-	Brassica oleracea	gemmifera	BRUSSELS SPROUT	EVESHAM GIANT	HRIGRU000342	C:01
ole13	*	-	Brassica oleracea	gemmifera	BRUSSELS SPROUT	WILHELMSBURGER	HRIGRU000605	C:01
ole14	*	yes	Brassica oleracea	wild	wild species C	C 04047	HRIGRU002075	C:04
ole15	*	-	Brassica oleracea	gemmifera	BRUSSELS SPROUT	SANDA ROEM VAN CASTRICUM	HRIGRU002227	C:01
ole16	*	-	Brassica oleracea	capitata	FODDER CABBAGE	CATTLE (EARLY DRUMHEAD)	HRIGRU002574	C:01
ole17	*	-	Brassica oleracea	gemmifera	BRUSSELS SPROUT	GROENENBOOM LATE SELECTION	HRIGRU002787	C:01
ole18	*	-	Brassica oleracea	botrytis	WINTER CAULIFLOWER	ST MALO HALF HATIF	HRIGRU002891	C:01
ole19	*	-	Brassica oleracea	unknown	B. oleracea	-	HRIGRU003317	C:01
ole2	*	yes	Brassica oleracea	alboglabra	Chinese kale	A12DHd	A12DHd	C:01
ole20	*	-	Brassica oleracea	italica	SPROUTING BROCCOLI	PURPLE SPROUTING LATE IMPROVED	HRIGRU003543	C:01
ole21	*	-	Brassica oleracea	italica	SPROUTING BROCCOLI	EARLY WHITE SPROUTING	HRIGRU003552	C:01
ole22	*	-	Brassica oleracea	acephala	BORECOLE KALE	WESTLAND WINTER VERDURA	HRIGRU003598	C:01
ole23	*	-	Brassica oleracea	unknown	B. oleracea	-	HRIGRU004250	C:01

ole24	*	-	Brassica oleracea	alboglabra	WHITE FLOWERED KALE	CHEMBERE DZAGUMHANA	HRIGRU004293	C:01
ole25	*	-	Brassica oleracea	botrytis	WINTER CAULIFLOWER	WINTER ROSCOFF	HRIGRU004492	C:01
ole26	*	-	Brassica oleracea	capitata	CABBAGE	ASPRO	HRIGRU004515	C:01
ole27	*	-	Brassica oleracea	unknown	B. oleracea	-	HRIGRU004515	C:01
ole28	*	-	Brassica oleracea	italica	CALABRESE	RAMOSO CALABRESE PRECOCE	HRIGRU004705	C:01
ole29	*	-	Brassica oleracea	capitata	SUMMER CABBAGE	CAVOLO CAPPUCCIO MEDIO NAPOLETANE	HRIGRU004771	C:01
ole3	*	yes	Brassica oleracea	gemmifera	Brussels sprout	AC498	AC498	C:01
ole30	*	-	Brassica oleracea	capitata	SAVOY CABBAGE	CAVOLO VERZA SAN GIOVANNI	HRIGRU004773	C:01
ole31	*	-	Brassica oleracea	botrytis	SUMMER CAULIFLOWER	BIANCO NAPOLETANE NATALINO	HRIGRU004814	C:01
ole32	*	-	Brassica oleracea	botrytis	WINTER CAULIFLOWER	GENARESE CAVOLFIORE	HRIGRU004821	C:01
ole33	*	-	Brassica oleracea	botrytis	AUTUMN CAULIFLOWER	DI JESI	HRIGRU004825	C:01
ole34	*	-	Brassica oleracea	botrytis	AUTUMN CAULIFLOWER	TOSCANO	HRIGRU004832	C:01
ole35	*	-	Brassica oleracea	botrytis	GREEN CAULIFLOWER	CIMA VERDE BARESE	HRIGRU004845	C:01
ole36	*	-	Brassica oleracea	botrytis	GREEN CAULIFLOWER	VERDE DI MACERATA	HRIGRU004847	C:01
ole37	*	-	Brassica oleracea	acephala	FODDER BLACK KALE	CAVOLO NERO DI TOSCANA O	HRIGRU004861	C:01
ole38	*	-	Brassica oleracea	italica	PURPLE HEAD BROCCOLI	DI SICILIA VIOLETTO	HRIGRU004872	C:01
ole39	*	-	Brassica oleracea	italica	BLACK BROCCOLI	BROCCOLO NERO SPIGARIELLO	HRIGRU004885	C:01
ole4	*	-	Brassica oleracea	acephala	kale	Arsis DH line	ARS 18	C:01
ole40	*	-	Brassica oleracea	gemmifera	BRUSSELS SPROUT	OLD BEDFORDSHIRE STOCK	HRIGRU005086	C:01
ole41	*	yes	Brassica oleracea	wild	wild species C	C 04048	HRIGRU005096	C:01
ole42	*	-	Brassica oleracea	botrytis	CAULIFLOWER	FANESE PRECOCE	HRIGRU005293	C:01
ole43	*	-	Brassica oleracea	gongylodes	KOHL RABI	CAVOLO	HRIGRU005389	C:01
ole44	*	yes	Brassica oleracea	italica	FEATHER LEAF BROCC	CAVOLO CAVOLINA RIZZA	HRIGRU005416	C:02
ole45	*	-	Brassica oleracea	italica	CALABRESE	BROCCOLO DI MOJO	HRIGRU005419	C:01
ole46	*	-	Brassica oleracea	italica	CALABRESE	CAVOLO BROCCOLO NATALINO	HRIGRU005425	C:01
ole47	*	-	Brassica oleracea	gongylodes	PURPLE KOHL RABI	CAVOLO FORTE	HRIGRU005443	C:01
ole48	*	-	Brassica oleracea	capitata	CABBAGE	SHETLAND CABBAGE	HRIGRU005652	C:01
ole49	*	-	Brassica oleracea	acephala	CURLY KALE	CURLY KALE	HRIGRU005693	C:01
ole5	*	-	Brassica oleracea	capitata	cabbage	Bohmerwaldkohl DH line 85c	BOH 85c	C:01
ole50	*	-	Brassica oleracea	botrytis	ROMANESCO CAULI	ROMANESCO NATALINO	HRIGRU006210	C:01
ole51	*	-	Brassica oleracea	gemmifera	BRUSSELS SPROUT	CAVOLO DI BRUXELLES MEZZO NANO	HRIGRU006212	C:01
ole52	*	-	Brassica oleracea	acephala	KALE	GIANT JERSEY KALE	HRIGRU006226	C:01
ole53	*	-	Brassica oleracea	botrytis	WINTER CAULIFLOWER	LATE QUEEN	HRIGRU006230	C:01
ole54	*	-	Brassica oleracea	acephala	KALE	RUSSIAN KALE	HRIGRU006234	C:01
ole55	*	-	Brassica oleracea	botrytis	AUTUMN CAULIFLOWER	TASMAN	HRIGRU006254	C:01
ole56	*	-	Brassica oleracea	acephala	KALE	HUNGRY GAP	HRIGRU006262	C:01

ole57	*	-	Brassica oleracea	acephala	Kale	New Zealand Rawara	HRIGRU006431	C:01
ole58	*	-	Brassica oleracea	capitata	CABBAGE	SEPTEMBER	HRIGRU006851	C:01
ole59	*	-	Brassica oleracea	wild	wild species C	C 04049	HRIGRU007218	C:01
ole6	*	-	Brassica oleracea	botrytis	Cauliflower	CA25	CA25	C:01
ole60	*	-	Brassica oleracea	wild	wild species C	C 04050	HRIGRU007234	C:01
ole61	*	-	Brassica oleracea	wild	wild species C	C 04051B	HRIGRU007319	C:01
ole62	*	-	Brassica oleracea	wild	wild species C	C 04052	HRIGRU007320	C:01
ole63	*	-	Brassica oleracea	wild	wild species C	C 04053	HRIGRU007321	C:01
ole64	*	-	Brassica oleracea	wild	wild species C	C 04054	HRIGRU007322	C:01
ole65	*	yes	Brassica oleracea	wild	wild species C	C 04055	HRIGRU007323	C:04
ole66	*	-	Brassica oleracea	wild	wild species C	C 04056	HRIGRU007338	C:01
ole67	*	-	Brassica oleracea	wild	wild species C	C 04057	HRIGRU007340	C:01
ole68	*	-	Brassica oleracea	wild	wild species C	C 04060	HRIGRU007343	C:01
ole69	*	-	Brassica oleracea	wild	wild species C	C 04061	HRIGRU007349	C:01
ole7	*	-	Brassica oleracea	acephala	kale	Butzo	CGN14111	C:01
ole70	*	-	Brassica oleracea	italica	BROCCOLI	BROCCOLO VERDE DI CALABRIA	HRIGRU007514	C:01
ole71	*	-	Brassica oleracea	alboglabra	CHINESE KALE	CHINESE KALE	HRIGRU007543	C:01
ole72	*	-	Brassica oleracea	acephala	KALE	FURCHENKOHL	HRIGRU007547	C:01
ole73	*	-	Brassica oleracea	wild	wild species C	C 04062	HRIGRU007795	C:01
ole74	*	-	Brassica oleracea	wild	WILD CABBAGE	-	HRIGRU007796	C:01
ole75	*	-	Brassica oleracea	wild	wild species C	C 04063	HRIGRU007796	C:01
ole76	*	-	Brassica oleracea	wild	wild species C	C 04064	HRIGRU007797	C:01
ole77	*	-	Brassica oleracea	capitata	TRONCHUDA CABBAGE	COUVE MURCIANA	HRIGRU007799	C:01
ole78	*	-	Brassica oleracea	capitata	JERSEY CABBAGE	-	HRIGRU007824	C:01
ole79	*	-	Brassica oleracea	acephala	KALE	-	HRIGRU008202	C:01
ole8	*	-	Brassica oleracea	italica	broccoli	Corvette DH line 12b	Cor12b	C:01
ole80	*	-	Brassica oleracea	gemmifera	BRUSSELS SPROUT	LOCAL SELECTION	HRIGRU008226	C:01
ole81	*	-	Brassica oleracea	botrytis	AUTUMN CAULIFLOWER	GIGANTE DI NAPOLI NATALINO	HRIGRU008558	C:01
ole82	*	yes	Brassica oleracea	wild	wild species C	C 04065	HRIGRU008694	C:01
ole83	*	-	Brassica oleracea	wild	wild species C	C 04066	HRIGRU008705	C:01
ole84	*	-	Brassica oleracea	wild	wild species C	C 04067	HRIGRU008707	C:01
ole85	*	-	Brassica oleracea	wild	wild species C	C 04068	HRIGRU008714	C:01
ole86	*	-	Brassica oleracea	wild	wild species C	C 04069	HRIGRU008724B	C:01
ole87	*	-	Brassica oleracea	tronchuda	TRONCHUDA KALE	COIVAO	HRIGRU009467	C:01
ole88	*	-	Brassica oleracea	tronchuda	TRONCHUDA CABBAGE	COUVE CORTE	HRIGRU009490	C:01
ole89	*	-	Brassica oleracea	tronchuda	TRONCHUDA CABBAGE	COUVE PENCA ASA DE CANTARO	HRIGRU009577	C:01
ole9	*	yes	Brassica oleracea	italica	Calabrese	GDDH33	GDDH33	C:01

ole90	*	-	Brassica oleracea	capitata	HYBRID CABBAGE	CAPE HORN (F1)	HRIGRU009836	C:01
ole91	*	-	Brassica oleracea	gongylodes	KOHL RABI	PURPLE VIENNA	HRIGRU011183	C:01
ole92	*	-	Brassica oleracea	capitata	WHITE CABBAGE	COUVE REPOLHO BACALAN	HRIGRU011490	C:01
ole93	*	-	Brassica oleracea	botrytis	WINTER CAULIFLOWER	NAPOLETANO MARZATICO	HRIGRU011732	C:01
ole94	*	-	Brassica oleracea	botrytis	ROMANESCO CAULI	ROMANESCO SAN GIUEPPE	HRIGRU011738	C:01
ole95	*	-	Brassica oleracea	unknown	B. oleracea	-	HRIGRU011846	C:01
ole96	*	-	Brassica oleracea	wild	wild species C	C 04097	LL25	C:01
ole97	*	-	Brassica oleracea	wild	wild species C	C 04096	LL3	C:01
ole98	*	-	Brassica oleracea	italica	broccoli	New River DH line 9b	Ner9b	C:01
ole99	*	-	Brassica oleracea	capitata	cabbage	China cabbage	PI436606	C:01
rap1		-	Brassica rapa	oleifera	RAPA OILSEED	CANDLE	CGN06482	A:01
rap10		-	Brassica rapa	rapa	TURNIP	INDO	HRIGRU 003116	A:01
rap11		-	Brassica rapa	nipposinica chinoleifera	JAPANESE GREENS	-	HRIGRU 004682	A:05
rap12		yes	Brassica rapa	ruvo	BROCOLETTO	-	HRIGRU 004714	A:05
rap13		yes	Brassica rapa	ruvo	BROCOLETTO	-	HRIGRU 004722	A:06
rap14		yes	Brassica rapa	ruvo	BROCOLETTO	-	HRIGRU 004734	A:06
rap15		-	Brassica rapa	pekinensis	CHINESE CABBAGE	NOZAKI EARLY	HRIGRU 005165	A:01
rap16		yes	Brassica rapa	ruvo	BROCOLETTO	-	HRIGRU 005274	A:05
rap17		yes	Brassica rapa	ruvo	BROCOLETTO	-	HRIGRU 005330	A:01
rap18		-	Brassica rapa	pekinensis	CHINESE CABBAGE	WONG BOK	HRIGRU 006143	A:01
rap19		-	Brassica rapa	pekinensis	CHINESE CABBAGE	-	HRIGRU 006175	A:01
rap2		-	Brassica rapa	oleifera	RAPA OILSEED	SOMALI SARISSA	CGN06837	A:04
rap20		-	Brassica rapa	pekinensis	CHINESE CABBAGE	-	HRIGRU 006202	A:01
rap21		yes	Brassica rapa	rapa	TURNIP	BELADI	HRIGRU 007204	A:04
rap22		-	Brassica rapa	chinensis	PAK CHOI	SI YUE MAN (BAI BANG)	HRIGRU 007569	A:01
rap23		-	Brassica rapa	chinensis	PAK CHOI	SI YUE MAN (QING BANG)	HRIGRU 007570	A:01
rap24		yes	Brassica rapa	chinensis	PAK CHOI	WU YUE MAN	HRIGRU 007573	A:01
rap25		-	Brassica rapa	rapa	TURNIP	-	HRIGRU 008170	A:01
rap26		-	Brassica rapa	rapa	TURNIP SHOOTS	NABAL DE GREILOS	HRIGRU 011606	A:04
rap27		yes	Brassica rapa	oleifera	RAPA OILSEED	KULTA	NGB2330	A:03
rap28		-	Brassica rapa	unknown	KHARDAL	KHARDAL	HRIGRU002413	A:03
rap29		yes	Brassica rapa	oleifera	RAPA OILSEED	DURO	NGB002698	A:01
rap3		-	Brassica rapa	oleifera	RAPA OILSEED	-	CGN07216	A:01
rap30		-	Brassica rapa	rapa	TURNIP	JAWA	HRIGRU003118	A:05
rap31		-	Brassica rapa	rapa	STUBBLE TURNIP	DEBRA	HRIGRU003253	A:01
rap32		-	Brassica rapa	rapa	STUBBLE TURNIP	LABRA	HRIGRU003254	A:02

rap33		yes	Brassica rapa	rapa	STUBBLE TURNIP	VOBRA	HRIGRU003256	A:04
rap34		-	Brassica rapa	rapa	TURNIP	CYCLON	HRIGRU003288	A:01
rap35		yes	Brassica rapa	rapa	STUBBLE TURNIP	APPIN	HRIGRU003302	A:05
rap36		-	Brassica rapa	rapa	STUBBLE TURNIP	CIVASTO	HRIGRU003339	A:01
rap37		-	Brassica rapa	rapa	STUBBLE TURNIP	TYFON	HRIGRU003341	A:01
rap38		yes	Brassica rapa	rapa	TURNIP	TUCKER'S CHAMPION GTY	HRIGRU003373	A:02
rap39		-	Brassica rapa	rapa	TURNIP	MARBLE TOP GREEN	HRIGRU003440	A:05
rap4		yes	Brassica rapa	chinensis	PAK CHOI	-	CGN07219	A:05
rap40		-	Brassica rapa	rapa	TURNIP	MILAN PURPLE TOP	HRIGRU003441	A:01
rap41		-	Brassica rapa	rapa	TURNIP	LONG D'ALSACE	HRIGRU004052	A:01
rap42		yes	Brassica rapa	ruvo	BROCOLETTO	Broccoletto o cima di rapa quarantino	HRIGRU004738	A:01
rap43		-	Brassica rapa	chinensis	Chinese Cabbage	Pak Choi	HRIGRU005107	A:01
rap44		-	Brassica rapa	nipposinica chinoleifera	JAPANESE GREENS	LATE KOMATSUNA	HRIGRU005163	A:05
rap45		-	Brassica rapa	rapa	TURNIP	COBRA	HRIGRU005180	A:04
rap46		yes	Brassica rapa	ruvo	BROCOLETTO	Broccoletto o cima di rapa cinquana	HRIGRU005248	A:05
rap47		-	Brassica rapa	wild	N/A	HRIGRU006699	HRIGRU006699	A:01
rap48		-	Brassica rapa	chinensis	PAK CHOI	PURPLE PAK CHOY	HRIGRU007542	A:01
rap49		-	Brassica rapa	rapa	TURNIP	DOLOMIT	HRIGRU007692	A:04
rap5		-	Brassica rapa	rapa	FODDER TURNIP	-	CGN07223	A:01
rap50		yes	Brassica rapa	wild	N/A	HRIGRU8688	HRIGRU008688	A:01
rap51		yes	Brassica rapa	wild	HRIGRU008689	HRIGRU008689	HRIGRU008689	A:05
rap52		yes	Brassica rapa	nipposinica chinoleifera	JAPANESE GREENS	MIZUNA (KYONA)	HRIGRU011277	A:05
rap53		-	Brassica rapa	rapa	STUBBLE TURNIP	BARKANT	HRIGRU012021	A:01
rap54		-	Brassica rapa	rapa	STUBBLE TURNIP	DISCO	HRIGRU012025	A:01
rap55		-	Brassica rapa	rapa	STUBBLE TURNIP	FORAGE STAR	HRIGRU012030	A:01
rap56		-	Brassica rapa	oleifera	RAPA OILSEED	DEBUT	HRIGRU013079	A:01
rap57		-	Brassica rapa	oleifera	RAPA OILSEED	AGENA	HRIGRU013109	A:04
rap58		-	Brassica rapa	rapa	STUBBLE TURNIP	RIVAL	HRIGRU013451	A:01
rap59		-	Brassica rapa	oleifera	RAPA OILSEED	FOCUS	HRIGRU013519	A:01
rap6		-	Brassica rapa	oleifera	RAPA OILSEED	-	CGN07225	A:01
rap60		-	Brassica rapa	oleifera	RAPA OILSEED	SALUT	HRIGRU013520	A:01
rap61		yes	Brassica rapa	oleifera	RAPA OILSEED	CHINOOK	HRIGRU013922	A:04
rap62		-	Brassica rapa	unknown	RAPE KALE (rapa!)	NABICA GRELEIRA	HRIGRU8679	A:01
rap63		yes	Brassica rapa	trilocularis	SARSON	-	PI165595	A:03
rap64		-	Brassica rapa	unknown	UNKNOWN	PI183664	PI183664	A:04
rap65		-	Brassica rapa	unknown	UNKNOWN	PI21 9904	PI219904	A:01

rap66		-	Brassica rapa	trilocularis	A genome rapa	R-O-18	R-0-18	A:03
rap67		-	Brassica rapa	pekinensis	CHINESE CABBAGE	-	VIR 911.139	A:05
rap68		yes	Brassica rapa	pekinensis	CHINESE CABBAGE	-	VIR 911.57	A:01
rap69		yes	Brassica rapa	pekinensis	CHINESE CABBAGE	-	VIR 911.58	A:01
rap7		yes	Brassica rapa	nipposinica chinoleifera	JAPANESE GREENS	Round leaved mibina	CGN17279	A:05
rap70		-	Brassica rapa	oleifera	RAPA OILSEED	-	VIR12	A:01
rap71		-	Brassica rapa	oleifera	RAPA OILSEED	-	VIR143	A:05
rap72		-	Brassica rapa	oleifera	RAPA OILSEED	-	VIR351	A:01
rap73		-	Brassica rapa	trilocularis	SARSON	-	VIR911.112	A:05
rap74		-	Brassica rapa	chinesis rosularis	CHINESE FLAT CABBAGE	-	VIR911.154	A:01
rap75		-	Brassica rapa	chinesis rosularis	CHINESE FLAT CABBAGE	-	VIR911.631	A:01
rap76		-	Brassica rapa	rapa	TURNIP	-	VIR98	A:01
rap77		-	Brassica rapa	parachinensis	CHOY SUM	CHOY SUM	HRIGRU 007575	A:01
rap78		yes	Brassica rapa	parachinensis	CHOY SUM	CHOY SUM	HRIGRU 007576	A:01
rap79		-	Brassica rapa		wild	Ouse	-	A:06
rap8		-	Brassica rapa	unknown	UNKNOWN	CR2240/88	CR2240/88	A:05
rap80		yes	Brassica rapa	-	wild	Cornwall	-	A:04
rap81		yes	Brassica rapa	-	wild	Cornwall	-	A:06
rap82		yes	Brassica rapa	-	wild	Avon Stratford	-	A:04
rap83		yes	Brassica rapa	-	wild	Nene Cogenhoe	-	A:06
rap84		yes	Brassica rapa	-	weedy	Primrose Hil	-	A:04
rap85		yes	Brassica rapa	-	wild	Nene Northampton	-	A:04
rap86		yes	Brassica rapa	-	wild	Ouse	-	A:01
rap87		yes	Brassica rapa	-	weedy	Primrose Hil	-	A:04
rap88		yes	Brassica rapa	-	wild	Nene Cogenhoe	-	A:04
rap89		yes	Brassica rapa	-	wild	Nene Cogenhoe	-	A:06
rap9		-	Brassica rapa	chinensis	PAK CHOI	PAK CHOY	HRIGRU 002488	A:01
rup1	*	-	Brassica rupestris	-	wild species C	C 04073	HRIGRU013131	C:09
rup2	*	-	Brassica rupestris	-	wild species C	C 04074	HRIGRU013132	C:09
rup3	*	-	Brassica rupestris	-	wild species C	C 04075	HRIGRU013133	C:08
rup4	*	-	Brassica rupestris	-	wild species C	C 04076	HRIGRU013139	C:01
vil1	*	-	Brassica villosa	-	wild species C	C 04083	HRIGRU007331	C:07
vil10	*	yes	Brassica villosa	bivoniana	wild species C	C 04089	HRIGRU013136	C:12
vil11	*	-	Brassica villosa	tinei	wild species C	C 04093	HRIGRU013137	C:09
vil12	*	-	Brassica villosa	tinei	wild species C	C 04094	HRIGRU013138	C:11
vil13	*	-	Brassica villosa	villosa	wild species C	C 04095	HRIGRU013143	C:01

vil2	*	-	Brassica villosa	draepanensis	wild species C	C 04090	HRIGRU012485	C:11
vil3	*	-	Brassica villosa	draepanensis	wild species C	C 04091	HRIGRU012701	C:11
vil4	*	-	Brassica villosa	draepanensis	wild species C	C 04092	HRIGRU013126	C:09
vil5	*	-	Brassica villosa	bivoniana	wild species C	C 04084	HRIGRU013127	C:09
vil6	*	-	Brassica villosa	bivoniana	wild species C	C 04085	HRIGRU013128	C:11
vil7	*	-	Brassica villosa	bivoniana	wild species C	C 04086	HRIGRU013130	C:12
vil8	*	-	Brassica villosa	bivoniana	wild species C	C 04087	HRIGRU013134	C:10
vil9	*	-	Brassica villosa	bivoniana	wild species C	C 04088	HRIGRU013135	C:12
