# Supplementary material for: Origins of the amphiploid species Brassica napus L. investigated by chloroplast and nuclear molecular markers
Source: BMC Plant Biol. 2010 Mar 29;10:54. doi: 10.1186/1471-2229-10-54 (PMC2923528; doi:10.1186/1471-2229-10-54)
Supplement: Additional file 2 — Table S2. Allelic constitution of the new chloroplast haplotypes detected in this study using the 6 chloroplast SSRs as well as those detected in samples used for the AFLP analysis [file 1471-2229-10-54-S2.PDF]

Table S2. Allelic constitution of the new chloroplast haplotypes detected in this study using the 6 chloroplast SSRs as well as those detected in samples used for the AFLP analysis

	Allele Size					
**Haplotype**	**Chla16**	**ChloroP**	**Chloro35**	**Chloro39**	**ChloroO**	**ChloroQ**
A:01	113	265	84	84	89	150
A:02	113	264	84	84	89	150
A:03	113	264	84	84	89	148
A:04	113	265	84	84	89	148
A:05	113	265	84	83	89	148
A:06	113	272	84	83	91	148
B:01	116	263	83	83	null	123
B:02	null	263	83	83	null	123
C:01	114	271	83	84	89	148
C:02	114	270	83	84	89	148
C:03	114	272	83	84	89	148
C:04	114	266	84	84	89	148
C:05	113	266	84	84	89	148
C:06	113	264	84	84	91	148
C:08	111	264	84	84	91	154
C:11	111	263	84	84	91	155
C:12	111	264	84	85	91	155
C:19	112	265	84	83	89	138
C:20	114	269	84	85	89	150
C:21	115	271	84	84	90	144
C:22	114	null	84	84	100	140
C:23	116	264	84	84	90	150
C:24	116	265	84	84	90	151
C:25	116	265	84	84	91	148
C:26	116	266	84	84	90	150
C:27	114	264	84	83	89	148
